# C-di-GMP regulates *Pseudomonas aeruginosa* stress response to tellurite during both planktonic and biofilm modes of growth

**DOI:** 10.1038/srep10052

**Published:** 2015-05-20

**Authors:** Song Lin Chua, Krishnakumar Sivakumar, Morten Rybtke, Mingjun Yuan, Jens Bo Andersen, Thomas E. Nielsen, Michael Givskov, Tim Tolker-Nielsen, Bin Cao, Staffan Kjelleberg, Liang Yang

**Affiliations:** 1 Singapore Centre on Environmental Life Sciences Engineering (SCELSE), Nanyang Technological University, Singapore 637551; 2 NUS Graduate School of Integrative Sciences and Engineering, National University of Singapore, Singapore 117543; 3 Interdisciplinary Graduate School, Nanyang Technological University, Singapore 637551; 4 Costerton Biofilm Center, Department of International Health, Immunology and Microbiology, University of Copenhagen, 2200 København N, Denmark; 5 School of Civil and Environmental Engineering, Nanyang Technological University, Singapore 639798; 6 Center for Marine Bio-Innovation and School of Biotechnology and Biomolecular Sciences, University of New South Wales, Sydney 2052, Australia; 7 School of Biological Sciences, Nanyang Technological University, Singapore 637551

## Abstract

Stress response plays an important role on microbial adaptation under hostile environmental conditions. It is generally unclear how the signaling transduction pathway mediates a stress response in planktonic and biofilm modes of microbial communities simultaneously. Here, we showed that metalloid tellurite (TeO_3_^2–^) exposure induced the intracellular content of the secondary messenger cyclic di-GMP (c-di-GMP) of *Pseudomonas aeruginosa*. Two diguanylate cyclases (DGCs), SadC and SiaD, were responsible for the increased intracellular content of c-di-GMP. Enhanced c-di-GMP levels by TeO_3_^2–^ further increased *P. aeruginosa* biofilm formation and resistance to TeO_3_^2–^. *P. aeruginosa* Δ*sadC*Δ*siaD* and PAO1/p_*lac*_-*yhjH* mutants with low intracellular c-di-GMP content were more sensitive to TeO_3_^2–^ exposure and had low relative fitness compared to the wild-type PAO1 planktonic and biofilm cultures exposed to TeO_3_^2–^. Our study provided evidence that c-di-GMP level can play an important role in mediating stress response in microbial communities during both planktonic and biofilm modes of growth.

Microorganisms display a striking ability to adapt to unfavorable conditions such as exposure to UV radiation, heavy metals and antibiotic treatments, by inducing stress responses and forming surface-attached biofilms^1^,^2^. Biofilms consist of microbial cells embedded in their self-produced extracellular polymeric substances (EPS). The EPS can account for up to 90% of the biofilm biomass and serve as physical barriers to protect biofilm cells^3^. Hence, biofilms dramatically increase the tolerance of bacterial cells towards environmental stress and immune attack during the course of infections^4^,^5^. Extensive intercellular communication and interactions have been observed within biofilms, and cells with distinct physiology may play different roles under stress conditions^6^,^7^,^8^.

Bis-(3′-5′)-cyclic dimeric guanosine monophosphate (C-di-GMP) plays an important role in biofilm formation of a wide range of bacteria^9^. Bacterial intracellular c-di-GMP content is determined by diguanylate cyclases (DGCs) that catalyze the formation of c-di-GMP and phosphodiesterases (PDEs), which degrade c-di-GMP^9^. When intracellular c-di-GMP content is high, bacterial cells reduce motility and increase synthesis of EPS matrix, resulting in biofilm formation^10^,^11^. In contrast, biofilm cells increase their motility and disperse from biofilms when the intracellular c-di-GMP content is low^12^,^13^. C-di-GMP signaling can be induced by stress conditions such as antimicrobial exposure^14^,^15^. The impact of c-di-GMP on mediating stress response by microbial communities during both planktonic and biofilm modes of growth remains unclear.

Anthropogenic activities have resulted in serious metal(loid) pollution, especially in industrialized countries and regions. The natural ecosystems are often direct or indirect recipients of toxic metal(loid)s such as TeO_3_^2−^. Many environmental bacteria including *Pseudomonas aeruginosa* are capable of surviving in the presence of TeO_3_^2−^ at low concentrations by reducing TeO_3_^2−^ to Te(0) nanomaterials, as a result of either detoxification, redox maintenance or respiration^16^,^17^,^18^,^19^. Although the toxic effects of metal(oild)s on environmental microorganisms at individual cell levels have been extensively studied^20^, little is known about the impacts of metal(loid)s on bacterial social behaviours^21^.

In the present study, we investigated the role of c-di-GMP in mediating stress responses by the opportunistic pathogen *Pseudomonas aeruginosa* to a toxic metalloid, tellurite (TeO_3_^2–^). TeO_3_^2−^ is highly toxic to most microbes and had been previously described by Alexander Fleming as an antimicrobial agent^22^. Bacterial cells take up TeO_3_^2–^ and subsequently reduce it to tellurium nanoparticles, which can be easily tracked by the black precipitates on the bacterial cell surface. Quantification of intracellular c-di-GMP and proteomic analysis indicated that c-di-GMP levels were induced by TeO_3_^2–^ exposure, which enhanced *P. aeruginosa* TeO_3_^2–^ resistance and biofilm formation. SadC and SiaD were found to be important in the induction of c-di-GMP by TeO_3_^2–^ exposure. We showed that mutants with low intracellular c-di-GMP content could be outcompeted by the wild-type strain from biofilm and planktonic cultures under metalloid stress condition.

## Results

### Stress responses of *P. aeruginosa* to TeO_3_^2−^ induced c-di-GMP signaling

**Figure 1:**
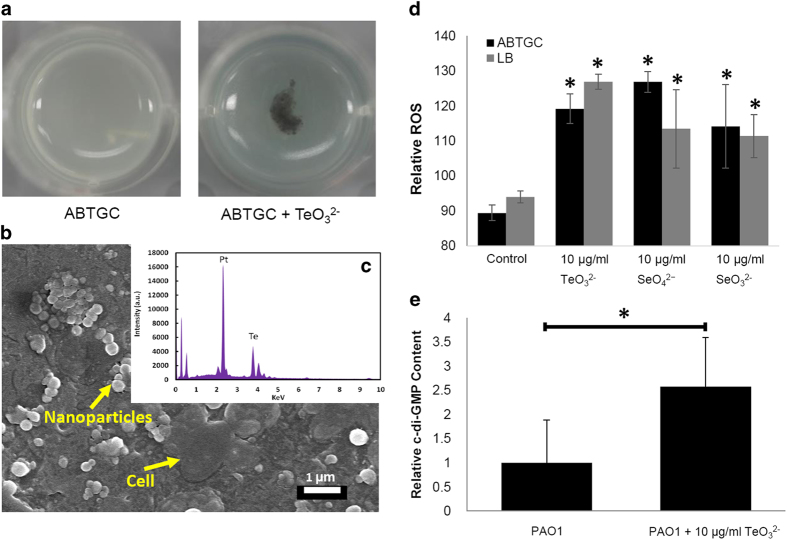
Aggregates formed by *P. aeruginosa* wild-type PAO1 in ABTGC medium with and without 10 μg/ml TeO_3_^2−^ under shaking condition after 1 d (**a**). Aggregates formed in TeO_3_^2−^ containing medium were analyzed by FE-SEM (**b**) and energy-dispersive X-ray spectroscopy (**c**). Arrows in the FE-SEM image indicate the bacterial cell and nanoparticles on the cell surface. ROS generation by *P. aeruginosa* PAO1 cells after exposure to sub-lethal concentration of TeO_3_^2−^ , SeO_3_^2−^ and SeO_4_^2−^ (**d**). Relative intracellular c-di-GMP content of PAO1 cultures in ABTGC medium with and without 10 μg/ml TeO_3_^2−^ was quantified by HPLC (**e**). Means and standard deviations of three replicates are shown. Student’s *t*-test was performed for testing differences between groups. * *P < 0.05*.

Cultivation of different bacterial species in the presence of sub-lethal concentrations of antimicrobial agents is a widely employed method to investigate their stress responses^23^,^24^,^25^. The MIC of *P. aeruginosa* to TeO_3_^2−^ is 100 μg/ml in ABTGC medium. Large aggregates (approximately 1-3 mm) were formed when *P. aeruginosa* was grown in ABTGC media containing 10 μg/ml TeO_3_^2−^ at 37 °C (Fig. 1a). Further analysis of the TeO_3_^2−^-induced aggregates by field emission scanning electron microscopy (FE-SEM) and energy-dispersive X-ray spectroscopy (EDX) revealed the presence of tellurium-containing precipitates around the bacterial cells (Fig. 1b,c). No tellurium-containing precipitates were observed for *P. aeruginosa* cells growing in medium without TeO_3_^2−^. Thus, the tellurium-containing precipitates might generate conditions of membrane-associated stress for *P. aeruginosa* cells.

TeO_3_^2−^ and oxyanions such as selenate/selenite are well known to exert their toxic effects on microorganisms via generation of reactive oxygen species (ROS)^26^,^27^. We measured the generation of ROS by *P. aeruginosa* cells exposed to sub-lethal concentrations of TeO_3_^2−^ as well as SeO_3_^2−^ and SeO_4_^2−^ by using the OxiSelect™ *in vitro* ROS/RNS assay kit. As anticipated, exposure of *P. aeruginosa* cells to the TeO_3_^2−^, SeO_3_^2−^ and SeO_4_^2−^ significantly increased their cytoplasmic ROS content regardless of the nutrient conditions (Fig. 1d).

### Proteomic analysis of TeO_3_^2−^ stressed *P. aeruginosa* cells

Oxidative stress response by *P. aeruginosa* leading to aggregate formation, recently reported to resemble the biofilm physiology^28^ has not been documented. We thus investigated the overall impact of TeO_3_^2−^ on *P. aeruginosa* cells using a comparative proteomic approach for cells cultivated with and without 10 μg/ml TeO_3_^2−^.

Using a p-value cut-off of 0.05 and a fold change cut-off of 5 (as described in the Materials and Methods), 129 proteins were significantly affected by TeO_3_^2−^ exposure with 64 proteins upregulated (Supplementary table 1) and 65 proteins being down-regulated (Supplementary table 2).

The expression of several of outer membrane associated proteins was induced by TeO_3_^2−^ treatment, including OprQ (PA2760, 28.8-fold), OprI precursor (PA2853, 15-fold), probable outer membrane protein precursor (PA2391, 10.9-fold), OprM (PA0427, 10.5-fold), OprL precursor (PA0973, 9.8-fold), OprD precursor (PA0958, 9.8-fold), OprB (PA3186, 9.7-fold) and OprC (PA3790, 8.1-fold) (Supplementary table 1). The membrane transporter CdrB of the large extracellular protein CdrA^29^ was induced 25.8-fold by exposure to TeO_3_^2−^ (Supplementary table 1). CdrAB expression has been used as a c-di-GMP indicator^30^ and reported to promote biofilm formation and auto-aggregation in a Psl polysaccharide dependent manner^29^, and co-immunoprecipitation experiments have clearly shown that CdrA binds to Psl^29^. HPLC analysis showed that *P. aeruginosa* PAO1 cultivated in ABTGC medium with 10 μg/ml TeO_3_^2−^ treatment had a higher relative intracellular c-di-GMP concentration compared to untreated control samples (approximately 2.5-fold) (Fig. 1e).

### SadC and SiaD contribute to c-di-GMP induction by TeO_3_^2−^

**Figure 2:**
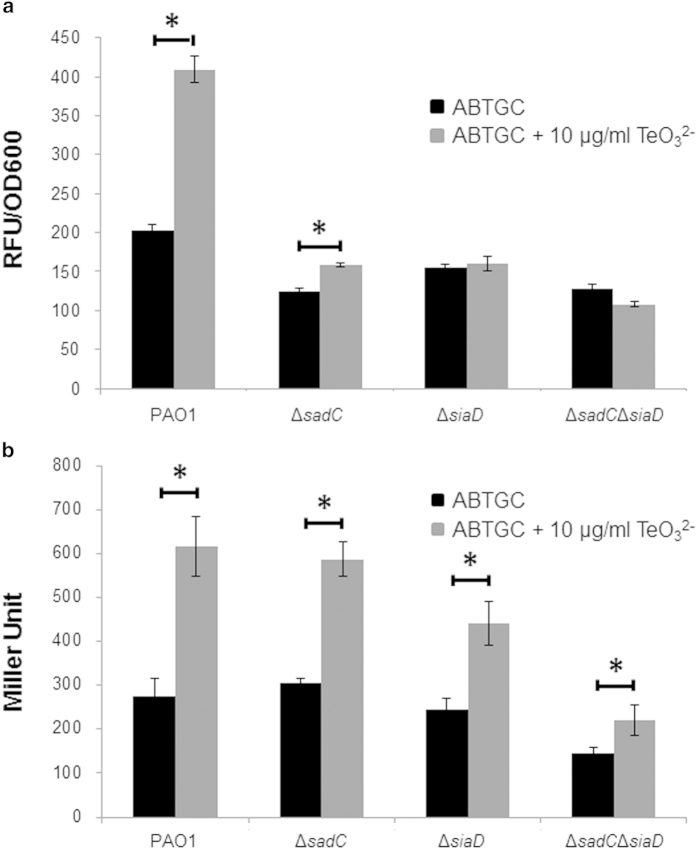
Expression of biosensor P_*cdrA*_-*gfp* (**a**) and P_*pel*_-*lacZ* (**b**) by *P. aeruginosa* strains in ABTGC medium with and without the presence of 10 μg/ml TeO_3_^2−^. The P_*cdrA*_*-gfp* expression was shown as relative fluorescence units (RFU) per OD_600_. The P_*pel*_-*lacZ* expression was shown as Miller Unit. Means and standard deviations of three replicates are shown. Student’s *t*-test was performed for testing differences between groups. * *P < 0.05*.

CdrAB belongs to a family of bacterial proteins secreted by the two-partner secretion system^31^. Recently, two other members of this family, XacFhaB from *Xanthomonas axonopodis* pv. Citri and FHA from *Bordetella pertussis* have also been implicated in biofilm formation^32^,^33^. These large inter-bacterial adhesins may play a key role in establishing structured biofilm communities under stress conditions. The *cdrA* promoter is positively regulated by the c-di-GMP concentration, and the expression of P_*cdrA*_*-gfp* has been recently used as a biosensor of the intracellular content of c-di-GMP in *P. aeruginosa*^30^. We tested the expression of the P_*cdrA*_*-gfp* reporter in *P. aeruginosa* cultures with and without the presence of TeO_3_^2−^ and found that TeO_3_^2−^ exposure significantly increased the expression of fluorescence in a dose dependent manner (Fig. 2a). This result is in accordance with our HPLC quantification and indicates that TeO_3_^2−^ exposure increases the intracellular content of c-di-GMP and that TeO_3_^2−^ induced aggregates might carry physiological traits similar to those of biofilms.

Recently, both SadC and SiaD, were shown to be able to transduce an extracellular signal generated by the toxic detergent SDS and catalyze synthesis of c-di-GMP for facilitating biofilm formation by *P. aeruginosa*^34^,^35^. The defect environmental signaling Δ*sadC* and Δ*siaD* mutants were severely impaired in expression of the P_*cdrA*_*-gfp* reporter in the presence of TeO_3_^2−^ (Fig. 2a). SiaD appears to be more important than SadC for P_*cdrA*_*-gfp* induction by TeO_3_^2−^ since the Δ*sadC* mutant still displayed a slight induction of P_*cdrA*_*-gfp* by TeO_3_^2−^ (Fig. 2a).

Exopolysaccharides are the major EPS components of *P. aeruginosa* biofilms and are well known to be induced by high intracellular c-di-GMP content in *P. aeruginosa*. We examined the expression of a *lacZ*-based biosensor of the Pel synthesis operon (mini-CTX-*pel*-*lacZ*^36^) in *P. aeruginosa* strains under TeO_3_^2−^ stress. As with P_*cdrA*_*-gfp* fusion, the expression of the *pel*-*lacZ* fusion was induced by TeO_3_^2−^ treatment, with SiaD essential for this induction (Fig. 2b). However, there was a slight induction of the *pel*-*lacZ* fusion by tellurite even in the Δ*sadC*Δ*siaC*D double mutant (Fig. 2b).

**Figure 3:**
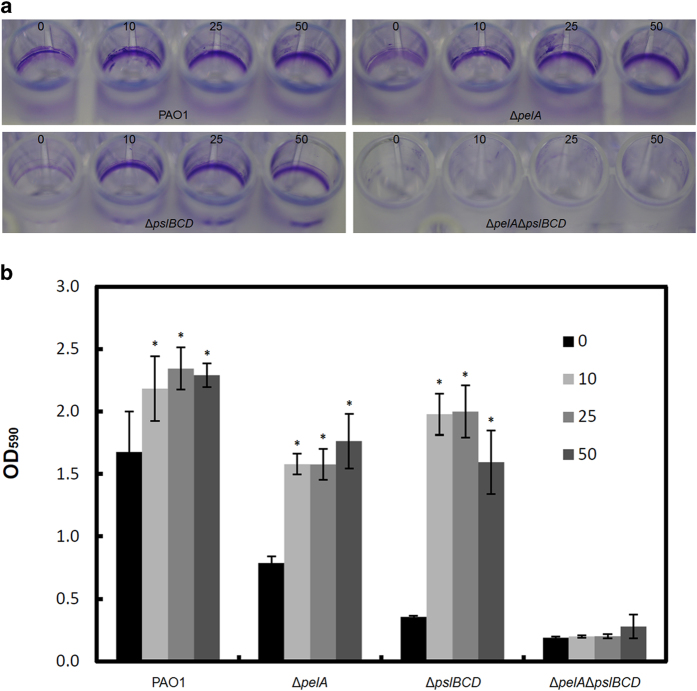
Biofilm formation by *P. aeruginosa* PAO1, Δ*pelA,* Δ*pslBCD* and Δ*pelA*Δ*pslBCD* in medium containing 0, 10, 25 and 50 μg/ml TeO_3_^2−^ under static conditions after 1 d incubation. Biofilms were firstly stained with 0.01% (w/v) crystal violet (**a**) and then quantified by dissolving in 96% ethanol and measuring absorbance at 590 nm (**b**). Means and standard deviations of three replicates are shown. Student’s *t*-test was performed for testing differences between groups. * *P < 0.05*.

Consistent with our observation of TeO_3_^2−^-induced aggregation, *P. aeruginosa* grown in the presence of TeO_3_^2−^ formed more biofilms than cells grown without TeO_3_^2−^ (Fig. 3). The induction of biofilm formation was dependent on the presence of Pel and Psl polysaccharides (Fig. 3).

### Induction of c-di-GMP confers a growth advantage under tellurite exposure during planktonic cultures

**Figure 4:**
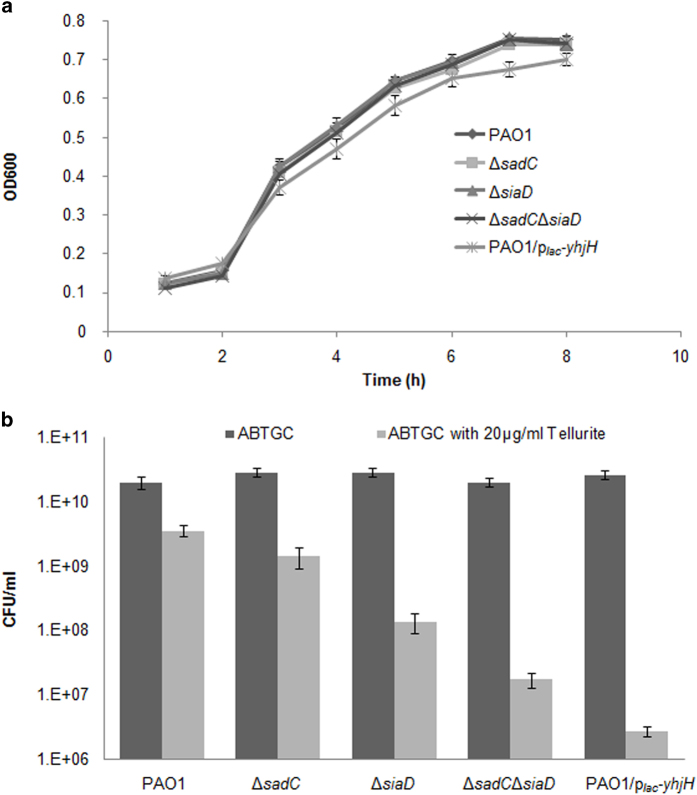
Growth curve (**a**) and TeO_3_^2−^ tolerance assay (**b**). *P. aeruginosa* PAO1, Δ*sadC*, Δ*siaD*, Δ*sadC*Δ*siaD*, and PAO1/p_*lac*_*-yhjH* strains were cultivated in ABTGC medium at 37 °C with shaking for growth measurement. For TeO_3_^2−^ tolerance assay, *P. aeruginosa* PAO1, Δ*sadC*, Δ*siaD*, Δ*sadC*Δ*siaD*, and PAO1/p_*lac*_*-yhjH* strains were cultivated in ABTGC medium with the presence of 20 μg/ml TeO_3_^2−^ overnight followed by CFU determination. Means and standard deviations of three replicates are shown.

Since c-di-GMP signaling was induced by TeO_3_^2−^ exposure, we examined whether induction of c-di-GMP signaling would confer a growth advantage of *P. aeruginosa* under TeO_3_^2−^ exposure. There was no growth defect of Δ*sadC*, Δ*siaD* and Δ*sadC*Δ*siaD* mutants under normal growth condition as compared to PAO1 control (Fig. 4a). However, the *P. aeruginosa* Δ*sadC*, Δ*siaD* single or double mutants were more sensitive to TeO_3_^2−^ (Fig. 4b). Similarly, the PAO1/p_*lac*_-*yhjH* mutant, which contains a PBBRMCS-2 plasmid with a constitutively expressed phosphodiesterase gene *yhjH* fused to and expressed by the *lac* promoter and thus has a low intracellular of c-di-GMP content^12^, was also more sensitive to TeO_3_^2−^ (Fig. 4). These results showed that intracellular c-di-GMP content determines the tolerance of *P. aeruginosa* to TeO_3_^2−^ exposure during planktonic cultures.

### Low intracellular c-di-GMP mutants lose fitness under stress during both planktonic and biofilm modes of growth

**Figure 5:**
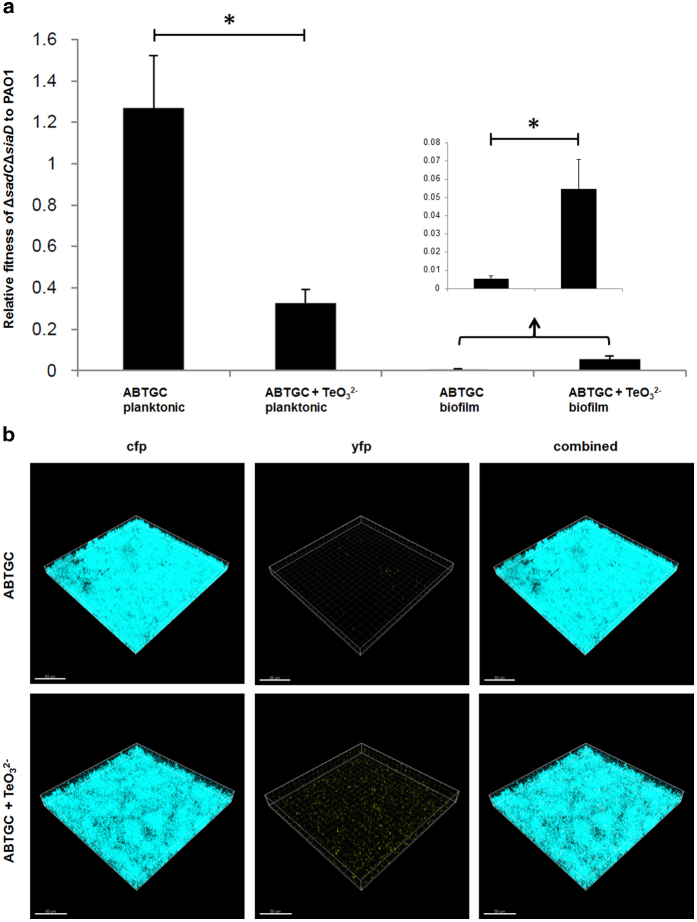
Relative fitness of Δ*sadC*Δ*siaD* mutant to PAO1 in planktonic co-cultures and biofilm co-cultures in ABTGC medium with and without the presence of 10 μg/ml TeO_3_^2−^ (**a**). Means and standard deviations of three replicates are shown. Student’s *t*-test was performed for testing differences between groups. * *P < 0.05*. CLSM images of biofilm co-cultures formed by cfp-tagged *P. aeruginosa* PAO1 and yfp-tagged Δ*sadC*Δ*siaD* mutant in ABTGC medium with and without the presence of 10 μg/ml TeO_3_^2−^ (**b**). Representative image from triplicate experiments was shown for each condition. Bars, 50 μm.

When *cfp*-tagged PAO1 and *yfp*-tagged Δ*sadC*Δ*siaD* mutant strains were combined 1:1 (vol/vol) for planktonic co-cultivation experiments, the wild-type showed higher survival rates and gained a higher level of relative fitness than the Δ*sadC*Δ*siaD* mutant in the presence of TeO_3_^2−^ than without TeO_3_^2−^ (Fig. 5a). Since diverse phenotypic and genotypic variants coexist in bacterial biofilms^37^,^38^, we tested whether TeO_3_^2−^ exposure-induced biofilm formation by high c-di-GMP containing cells would lead to protection of mutants with low intracellular c-di-GMP content in co-cultures. Here, PAO1 displayed a higher relative fitness than the Δ*sadC*Δ*siaD* mutant in biofilm co-cultures with and without the presence of TeO_3_^2−^ (Fig. 5b). However, the relative fitness of Δ*sadC*Δ*siaD* compared to PAO1 in biofilm co-cultures was slightly higher with the presence of TeO_3_^2−^ than in its absence (Fig. 5b). This suggests TeO_3_^2−^ could potentially induce expression of other DGC harboring proteins in the Δ*sadC*Δ*siaD* mutant and partly restore the intracellular c-di-GMP levels and biofilm formation.

**Figure 6:**
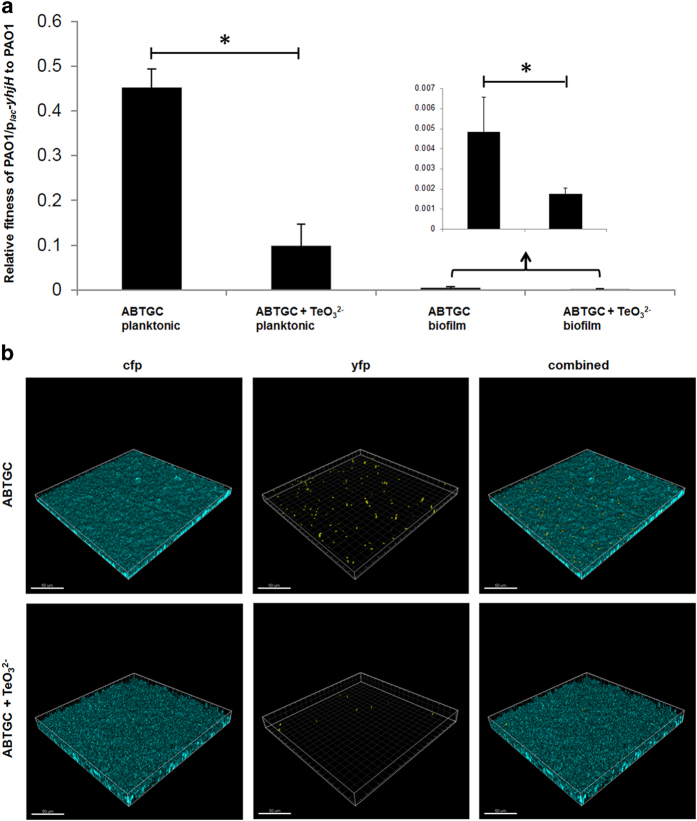
Relative fitness of PAO1/p_*lac*_*-yhjH* mutant to PAO1 in planktonic co-cultures and biofilm co-cultures in ABTGC medium with and without the presence of 10 μg/ml TeO_3_^2−^ (**a**). Means and standard deviations of three replicates are shown. Student’s *t*-test was performed for testing differences between groups. * *P < 0.05*. CLSM images of biofilm co-cultures formed by cfp-tagged *P. aeruginosa* PAO1 and yfp-tagged PAO1/p_*lac*_*-yhjH* mutant in ABTGC medium with and without the presence of 10 μg/ml TeO_3_^2−^ (**b**). Representative image from triplicate experiments was shown for each condition. Bars, 50 μm.

When we mixed *cfp*-tagged PAO1 and *yfp*-tagged PAO1/p_*lac*_-*yhjH* strains 1:1 (vol:vol) for planktonic co-cultivation experiments, the wild-type PAO1 strain gained a higher level of relative fitness than the c-di-GMP depleted PAO1/p_*lac*_-*yhjH* strain with and without exposure to TeO_3_^2−^ (Fig. 6a). Moreover, PAO1/p_*lac*_-*yhjH* was fully outcompeted by PAO1 in biofilm co-cultures supplemented with TeO_3_^2−^ (Fig. 6b). These results suggest that variants with low intracellular c-di-GMP content are unlikely to be protected and maintained by both *P. aeruginosa* planktonic and biofilm communities when c-di-GMP is required for stress response.

## Discussion

Bacterial cells face various types of stress during the colonization of natural environments and hosts. A series of stress response mechanisms has evolved in bacteria to cope with these harmful conditions. One well characterized stringent stress response mechanism is SpoT-mediated ppGpp accumulation, which can be provoked by nutritional stress caused by harmful conditions such as antibiotic treatment and UV irradiation^39^. ppGpp is able to bind directly to the bacterial RNA polymerase and further regulate transcriptional activity of many genes.

In addition to the stringent stress response, bacteria employ a wide range of social behaviors for surviving under unfavorable environmental conditions and these responses also contribute to bacterial pathogenesis^40^. For example, the *Staphylococcus aureus* agr quorum-sensing system is involved in the oxidative stress response^41^. Biofilm formation is evoked as a stress response mechanism by a wide range of bacteria^42^. It involves encasing bacterial cells inside the densely packed EPS matrix components and attaching firmly to biotic and abiotic surfaces. Biofilms are up to 1,000 times more resistant to antimicrobial agents compared to their planktonic counterparts^43^.

Recently, bacteria were found to form floating biofilm-resembling aggregates that are resistant to antimicrobials and phagocytosis^28^. Our work here showed that TeO_3_^2−^ exposure can elevate the c-di-GMP level in *P. aeruginosa* and lead to the formation of floating aggregates. TeO_3_^2−^-induced floating aggregate formation requires Pel and Psl polysaccharides as well as extracellular DNA (eDNA) (Fig. S1), in accordance with the Psl polysaccharide-eDNA interaction enabling the formation of skeleton of *P. aeruginosa* biofilms^44^. In addition to serving as matrix scaffolds, the polysaccharides could also induce synthesis of iron siderophore pyoverdine via the Gac/Rsm pathway in the floating aggregates, as we had previously demonstrated^45^. The formation of stress-induced biofilm-resembling aggregates might contribute to the dissemination of infection in the host.

The results presented here demonstrate that *P. aeruginosa* mutants with low c-di-GMP content were more sensitive to TeO_3_^2−^ exposure in planktonic cultures and thus their growth was negatively affected by TeO_3_^2−^ exposure, as compared to c-di-GMP containing wild-type strain (Fig. 4). Consistent with this finding, a recent study on biodegradation of 3-chloroaniline by *Comamonas testosteroni* reported that, compared with the wild type, the strain with an elevated c-di-GMP level exhibited a better growth on the toxic substrate at high concentrations^46^. In addition to TeO_3_^2−^, the detergent Na-dodecylsulfate (SDS)^35^ also raised the c-di-GMP levels and caused aggregation of *P. aeruginosa*. In accordance with the TeO_3_^2−^ findings, the Δ*siaD* mutant with low intracellular c-di-GMP content was more sensitive to SDS during planktonic growth^35^. Together, these studies highlight that c-di-GMP signaling is involved in multiple stress response mechanisms, which might due to multiple DGCs and PDEs being encoded by many bacterial species.

Finally, we found that wild-type PAO1 strain biofilms prevented the attachment of mutants with low intracellular c-di-GMP content in both normal and TeO_3_^2−^ stress co-cultures. Our previous study revealed that the polysaccharides in *P. aeruginosa* biofilms could not be shared, for structural or functional benefits, by mutants that are defective in their synthesis^38^. These latter findings corroborate with the results presented here, and c-di-GMP mediated synthesis of polysaccharides may form another strategy to repress the proliferation and maintenance of c-di-GMP defective variants in biofilms. Considering that polysaccharides with similar structure to the *P. aeruginosa* polysaccharides are widely distributed in natural bacterial species, our results might reflect a conserved strategy employed by a range of bacterial species to repress the spreading of variants which cannot respond to environmental conditions by moderating their own c-di-GMP levels.

## Methods

### Bacterial strains and growth medium

The bacterial strains, plasmids, and primers used in this study are listed in Table 1. *Escherichia coli* DH5α strain was used for standard DNA manipulations. LB medium^47^ was used to cultivate *E. coli* strains. Batch cultivation of *P. aeruginosa* was carried out at 37 °C in ABT minimal medium^7^ supplemented with 5 g glucose l^–1^ (ABTG) or 2 g glucose l^–1^ and 2 g casamino acids l^–1^ (ABTGC). For plasmid maintenance in *E. coli*, the medium was supplemented with 100 μg ampicillin (Ap) ml^−1^, 15 μg gentamicin (Gm) ml^−1^, 15 μg tetracycline (Tc) ml^−1^, or 8 μg chloramphenicol (Cm) ml^−1^. For marker selection in *P. aeruginosa*, 30 μg Gm ml^−1^, 50 μg Tc ml^−1^, and 200 μg carbenicillin (Cb) ml^−1^ were used, when appropriate. Antibiotics were not added to *P. aeruginosa* cultures for c-di-GMP, stress response and biofilm assays as the plasmids we used were highly stable for these short-term experiments.

### Construction of *P. aeruginosa* mutants.

The Δ*pelA*, Δ*pslBCD* and Δ*pelA*Δ*pslBCD* mutants defective for Pel and/or Psl polysaccharide biogenesis were constructed by allelic displacement as previously described^48^. The Δ*sadC*, Δ*siaD* and Δ*sadC*Δ*siaD* mutants defective for SadC and/or SiaD diguanylate cyclase were constructed by allelic displacement as previously described^34^.

### Quantification of static biofilms

The microtitre tray biofilm formation assay was performed as described by O’Toole & Kolter^49^. Briefly, overnight cultures grown in ABTG medium were diluted to OD_600_ = ~0.001 with fresh ABTG medium and transferred to the wells of polystyrene 96-well microtitre trays (200 μl per well) and incubated for 24 h at 37 °C. Liquid culture was removed from each well and the wells were washed twice with 0.9% NaCl followed by staining with 0.1% crystal violet and washing twice with 0.9% NaCl. The crystal violet-stained biofilms were then resuspended in 96% ethanol, and the absorbance of biofilm-associated dye was measured at 600 nm. Experiments were performed in triplicate, and the results are shown as the mean ± sd.

### Field emission scanning electron microscopy (FE-SEM) and energy-dispersive X-ray spectroscopy (EDX)

The aggregates were dried and coated with platinum (Pt) using a vacuum electric sputter coater (JEOL JFC-1300, JEOL Asia Pte Ltd, Singapore). SEM images were taken using a field emission scanning electron microscope (FE-SEM, JSM-7600, JEOL Asia Pte Ltd, Singapore) at a voltage of 2.0-5.0 kV and EDX spectra were obtained using an energy-dispersive X-ray spectroscope (AZtecEnergy, Oxford Instruments, Oxfordshire, UK) as previously described^50^. Experiments were performed in triplicate, and representative images were shown.

### Reactive oxygen species (ROS) assay

PAO1 cultures were grown in ABTGC or LB medium controls and media with 10 μg ml^−1^ TeO_3_^2−^, SeO_3_^2−^ and SeO_4_^2−^, respectively. The ROS content of 1 ml stationary phase bacterial cells were then measured by using the OxiSelect™ *in vitro* ROS/RNS assay kit (Green Fluorescence), accordingly to manufacturer’s instructions. 2’, 7’-dichlorodihydrofluorescein (DCF) was used as a standard and the concentrations of ROS from PAO1 cultures were estimated according to the DCF standard curve. The fluorescence of the samples was read by the Tecan Infinite 2000 Microplate Reader at 480 nm excitation/530 nm emission. Experiments were performed in triplicate, and the results are shown as the mean ± sd. Student’s *t*-test was performed for testing differences between groups.

### iTRAQ-based proteomics analyses

*P. aeruginosa* PAO1 was grown in ABTG medium with and without 10 μg/ml TeO_3_^2−^ at 37 °C with shaking until stationary phase was reached. Cells were harvested and iTRAQ-based proteomics analyses were carried out as previously described^12^.

### Determination of minimal inhibitory concentration (MIC)

The MIC assays employed a microtiter broth dilution method as previously described in the NCSLA guidelines^51^. Briefly, fresh ~16 h cultures of *P. aeruginosa* were diluted in ABTG medium. For determination of MIC, potassium tellurite was dissolved in water at a concentration 10 times higher than the required range by serial dilutions from a stock solution. 10 μl of each concentration were added to each corresponding well of a 96-well microtiter plate (polypropylene, Costar Corp.) and 90 μl of bacterial culture (~1 × 10^5^ cells) in ABTG medium were added. The plate was incubated at 37 °C for 16-18 h. MIC was taken as the lowest concentration where no visual growth (based on OD_600_) of bacteria was detected. Experiments were performed in triplicate and representative results were shown.

### TeO_3_^2−^ tolerance assay

Overnight cultures of different *P. aeruginosa* strains were inoculated into ABTGC medium containing 20 μg/ml TeO_3_^2−^ and cultivated overnight (24 h). Overnight cultures were serially diluted and plated onto LB agar media. LB plates were incubated at 37 °C overnight before CFU calculation. Experiments were performed in triplicate, and the results are shown as the mean ± sd.

### Beta-galactosidase activity assay

A classical β-galactosidase assay^52^ was used to measure expression of the P_*pel*_*-lacZ* fusion in *P. aeruginosa* strains transformed with the mini-CTX-*pel*-lacZ fusion^36^, which carries the *pel* promoter fused to the *E. coli lacZ* gene. Experiments were performed in triplicate, and the results are shown as the mean ± sd. Student’s *t*-test was performed for testing differences between groups.

### Gfp reporter fusion assay

The expression of the c-di-GMP P_*cdrA*_*-gfp* biosensor^30^ in *P. aeruginosa* strains in the presence and absence of TeO_3_^2−^ was monitored by using a Tecan Infinite 2000 Microplate Reader. Monitoring strains were cultivated in 24-well microtiter plate with ABTGC medium with different concentrations of TeO_3_^2−^ at 37 °C with shaking. OD_600_ and GFP fluorescence (in relative fluorescence units, rfu) were measured every hour until the culture reach stationary growth phase. Experiments were performed in triplicate, and the results are shown as the mean ± sd. Student’s *t*-test was performed for testing differences between groups.

### Quantification of c-di-GMP concentration

Extraction of c-di-GMP was conducted as previously described^45^. 10 ml of *P. aeruginosa* cells in the early stationary phase from the ABTGC medium with and without 10 μg/ml TeO_3_^2−^ were washed twice with 1 mM ammonium acetate. Cells were lysed with 0.6 M HClO_4_ on ice for 30 min. Cell debris was removed by centrifugation and supernatant was neutralized to pH 6.0 with the addition of 2.5 M KHCO_3_. The precipitated KClO_4_ was removed by centrifugation and the supernatant was used for relative quantification of c-di-GMP. The concentration was measured by High Performance Liquid Chromatography (HPLC), the injection volume is 20 µl with 254 nm as detection wavelength. Reverse-phase C18 Targa column (2.1 x 40 mm, 5 μm) (catalog number: TR-0421-C185) was used with solvent A (10 mM ammonium acetate in water) and solvent B (10 mM ammonium acetate in methanol) at a flow rate of 0.2 ml min-1. Eluent gradient is as follows: 0 to 8 min, 1% B; 8 to 14 min, 15% B; 14 to 16 min, 19% B; 16 to 24 min, 100% B; 24 to 32 min, 100% B; 32 to 40 min, 1% B; 40 to 42 min, 1% B. The retention time of c-di-GMP is around 14.0 min. The c-di-GMP concentration was normalized by total protein concentration. The relative c-di-GMP concentrations of cells treated with 10 μg ml^−1^ tellurite against cells in ABTGC only were shown. Experiments were performed in triplicate, and the results are shown as the mean ± sd. Student’s *t*-test was performed for testing differences between groups.

### Competition assay

Competition assays were performed in both planktonic and biofilm co-cultures. In planktonic co-cultures, *cfp*-tagged wild-type PAO1 was mixed 1:1 (vol/vol) with *yfp*-tagged PAO1/p_*lac*_-*yhjH* (or *yfp*-tagged Δ*sadC*Δ*siaD*) and the mixtures inoculated into fresh ABTGC medium with and without the presence of 10 μg/ml TeO_3_^2−^. For relative fitness calculation, co-cultures were plated in LB agar plates after 24 h cultivation at 37 °C with shaking. Colony-forming units (CFUs) *N*_*i*_ were determined from three individual experiments and the number of PAO1 and PAO1/p_*lac*_-*yhjH* (or Δ*sadC*Δ*siaD*) colonies were determined based on their specific fluorescence at times *t *= 0 and at *t *= *T*. Relative fitness was determined as *r*_*ij *_= [*N*_*i*_(*T*)-*N*_*i*_(0)]/[*N*_*j*_(*T*)-*N*_*j*_(0)] as previously described with modification^53^, resulting in a fitness of ‘1’ when competing organisms are equally fit. Experiments were performed in triplicate, and the results are shown as the mean ± sd. Student’s *t*-test was performed for testing differences between groups.

In biofilm co-cultures, *cfp*-tagged wild-type PAO1 cells were mixed with *yfp*-tagged PAO1/p_*lac*_-*yhjH* (or *yfp*-tagged Δ*sadC*Δ*siaD*) cells at 1:1 (vol/vol) and the mixtures were inoculated into fresh ABTGC medium with and without the presence of 10 μg/ml TeO_3_^2−^. Static biofilms were cultivated on cover slides at 37 °C for 24 h as previously described^54^. Biofilms were imaged with a Zeiss LSM780 confocal laser scanning microscope (CLSM) equipped with detectors and filter sets for monitoring of Cfp and Yfp fluorescence. Images were obtained using a 40 × /1.4 objective. Simulated three-dimensional images and sections as well as biovolumes were generated using the Imaris software package (Bitplane AG)^8^. The biovolume *V*_*i*_ of each strain in the biofilm mode was determined from three individual experiments based on their fluorescence at times *t *= 0 and at *t *= *T*. Relative fitness was determined as *r*_*ij *_= [*V*_*i*_(*T*)-*V*_*i*_(0)]/[*V*_*j*_(*T*)-*V*_*j*_(0)] as previously described with modification^53^, resulting in a fitness of ‘1’ when competing organisms are equally fit. Experiments were performed in triplicate, and the results are shown as the mean ± sd. Student’s *t*-test was performed for testing differences between groups.

## Author Contributions

T.T.N., B.C., S.K. and L.Y. designed the project. S.L.C., M.T.R., J.B.A., M.J.Y. and K.S. performed the experiments. T.E.N., M.G., B.C. and L.Y. interpreted data. B.C. and L.Y. wrote the main manuscript text. All authors reviewed the manuscript.

## Additional Information

**How to cite this article**: Chua, S. L. *et al.* C-di-GMP regulates *Pseudomonas aeruginosa* stress response to tellurite during both planktonic and biofilm modes of growth. *Sci. Rep.* **5**, 10052; doi: 10.1038/srep10052 (2015).

## Supplementary Material

Supplementary Information

## Figures and Tables

**Table 1:** Strains and plasmids used in this study.

**Strain(s) or plasmid**	**Relevant characteristic(s)**	**Source or reference**
*P. aeruginosa* strains		
PAO1	Prototypic wild-type strain	55
Δ*pelA*	Gm^r^; *pelA* derivative of PAO1 constructed by allelic exchange	38
Δ*pslBCD*	Gm^r^; *pslBCD* derivative of PAO1 constructed by allelic exchange	38
Δ*pelA*Δ*pslBCD*	Gm^r^; *pelA*/*pslBCD* derivative of PAO1 constructed by allelic exchange	38
Δ*sadC*	Gm^r^; *sadC* derivative of PAO1 constructed by allelic exchange	This study
Δ*siaD*	Gm^r^; *siaD* derivative of PAO1 constructed by allelic exchange	This study
Δ*sadC*Δ*siaD*	Gm^r^; *sadC*/*siaD* derivative of PAO1 constructed by allelic exchange	This study
PAO1/p_*cdrA*_*-gfp*	Gm^r^; PAO1 carrying the p_*cdrA*_*-gfp* report	30
Δ*sadC*/p_*cdrA*_*-gfp*	Gm^r^; Δ*sadC* carrying the p_*cdrA*_*-gfp* report	This study
Δ*siaD*/p_*cdrA*_*-gfp*	Gm^r^; Δ*siaD* carrying the p_*cdrA*_*-gfp* report	This study
Δ*sadC*Δ*siaD*/p_*cdrA*_*-gfp*	Gm^r^; Δ*sadC*Δ*siaD* carrying the p_*cdrA*_*-gfp* report	This study
PAO1/p_*lac*_*-yhjH*	Tc^r^; PAO1 containing the p_*lac*_*-yhjH* vector	12
PAO1/p_*pel*_*-lacZ*	Tc^r^; PAO1 carrying the mini-CTX-*pelA*-*lacZ* report	This study
Δ*sadC*/p_*pel*_*-lacZ*	Tc^r^; Δ*sadC* carrying the mini-CTX-*pelA*-*lacZ* report	This study
Δ*siaD*/p_*pel*_*-lacZ*	Tc^r^; Δ*siaD* carrying the mini-CTX-*pelA*-*lacZ* report	This study
Δ*sadC*Δ*siaD*/p_*pel*_*-lacZ*	Tc^r^; Δ*sadC*Δ*siaD* carrying mini-CTX-*pelA*-*lacZ* report	This study
*E. coli* strain		
DH5α	F^–^, ø80d*lacZ*ΔM15, Δ(*lacZYA-argF*)U169, *deo*R, *recA*1, *endA*1, *hsdR*17(rK^−^, mK^+^), *phoA*, *supE*44, λ–, *thi*-1, *gyrA*96, *relA*1	Labotorary collection
*Plasmids*		
pUCP22	Ap^r^; Gm^r^; Broad-host-range cloning vector	56
pMPELA	Ap^r^; Gm^r^; *pelA* allelic replacement vector	57
pMPSL-KO1	Ap^r^; Gm^r^; *pslBCD* allelic replacement vector	58
pEX18Gm::Δ*sadC*	Gm^r^; *sadC* allelic replacement vector	34
pEX18Gm::Δ*siaD*	Gm^r^; *siaD* allelic replacement vector	34
pFLP2	Ap^r^; Source of FLP recombinase	59
p_*cdrA*_*-gfp*	Ap^r^; Gm^r^; pUCP22 carrying the p_cdrA_*-gfp* fusion	30
pRK600	Cm^r^; *ori* ColE1 RK2-Mob^+^ RK2-Tra^+^; helper vector for conjugation	60
p_*lac*_-*yhjH*	Tc^r^; pBBR1MCS3 carrying the *yhjH* gene	12
Mini-CTX-*pel*-lacZ	Tc^r^; mini-CTX vector carrying the *pel*-*lacZ* fusion	36

## References

[b1] Schwartz T., Hoffmann S. & Obst U. Formation of natural biofilms during chlorine dioxide and u.v. disinfection in a public drinking water distribution system. J Appl Microbiol. 95, 591–601 (2003). 12911708 10.1046/j.1365-2672.2003.02019.x

[b2] Baker-Austin C., Wright M. S., Stepanauskas R. & McArthur J. V. Co-selection of antibiotic and metal resistance. Trends Microbiol. 14, 176–182 (2006). 16537105 10.1016/j.tim.2006.02.006

[b3] Flemming H. C., Neu T. R. & Wozniak D. J. The EPS matrix: the “house of biofilm cells”. J Bacteriol 189, 7945–7947 (2007). 17675377 10.1128/JB.00858-07 PMC2168682

[b4] Stewart P. S. & Costerton J. W. Antibiotic resistance of bacteria in biofilms. Lancet 358, 135–138 (2001). 11463434 10.1016/s0140-6736(01)05321-1

[b5] Yang L. *et al.* Combating biofilms. FEMS Immunol Med Microbiol 65, 146–157 (2012). 22066868 10.1111/j.1574-695X.2011.00858.x

[b6] Herrmann G. *et al.* Colistin-tobramycin combinations are superior to monotherapy concerning the killing of biofilm *Pseudomonas aeruginosa*. J Infect Dis. 202, 1585–1592, 10.1086/656788 (2010). 20942647

[b7] Yang L. *et al.* Effects of iron on DNA release and biofilm development by *Pseudomonas aeruginosa*. Microbiology 153, 1318–1328 (2007). 17464046 10.1099/mic.0.2006/004911-0

[b8] Chua S. L. *et al.* Dispersed cells represent a distinct stage in the transition from bacterial biofilm to planktonic lifestyles. Nature communications 5, 4462 (2014). 10.1038/ncomms5462 25042103

[b9] Hengge R. Principles of c-di-GMP signalling in bacteria. Nat Rev Microbiol. 7, 263–273 (2009). 19287449 10.1038/nrmicro2109

[b10] Lory S., Merighi M. & Hyodo M. Multiple activities of c-di-GMP in Pseudomonas aeruginosa. Nucleic Acids Symp Ser (Oxf) 53, 51–52 (2009). 10.1093/nass/nrp026 19749255

[b11] Yang L. *et al.* Pattern differentiation in co-culture biofilms formed by Staphylococcus aureus and *Pseudomonas aeruginosa*. FEMS Immunol Med Microbiol. 62, 339–347 (2011). 21595754 10.1111/j.1574-695X.2011.00820.x

[b12] Chua S. L. *et al.* Bis-(3’-5’)-cyclic dimeric GMP regulates antimicrobial peptide resistance in *Pseudomonas aeruginosa*. Antimicrob Agents Chemother 57, 2066–2075 (2013). 23403434 10.1128/AAC.02499-12 PMC3632963

[b13] Gjermansen M., Nilsson M., Yang L. & Tolker-Nielsen T. Characterization of starvation-induced dispersion in *Pseudomonas putida* biofilms: genetic elements and molecular mechanisms. Mol Microbiol. 75, 815–826 (2010). 19602146 10.1111/j.1365-2958.2009.06793.x

[b14] Kaplan J. B. Antibiotic-induced biofilm formation. Int J Artif Organs 34, 737–751 (2011). 22094552 10.5301/ijao.5000027

[b15] Klebensberger J., Lautenschlager K., Bressler D., Wingender J. & Philipp B. Detergent-induced cell aggregation in subpopulations of *Pseudomonas aeruginosa* as a preadaptive survival strategy. Environ Microbiol. 9, 2247–2259 (2007). 17686022 10.1111/j.1462-2920.2007.01339.x

[b16] Klonowska A., Heulin T. & Vermeglio A. Selenite and tellurite reduction by *Shewanella oneidensis*. Appl Environ Microbiol. 71, 5607–5609 (2005). 16151159 10.1128/AEM.71.9.5607-5609.2005 PMC1214622

[b17] Yurkov V., Jappé J. & Verméglio A. Tellurite resistance and reduction by obligately aerobic photosynthetic bacteria. Appl Environ Microbiol. 62, 4195–4198 (1996). 16535446 10.1128/aem.62.11.4195-4198.1996 PMC1388984

[b18] Moscoso H., Saavedra C., Loyola C., Pichuantes S. & Vasquez C. Biochemical characterization of tellurite-reducing activities of Bacillus stearothermophilus V. Res Microbiol. 149, 389–397 (1998). 9766238 10.1016/s0923-2508(98)80321-5

[b19] Trutko S. M. *et al.* Involvement of the respiratory chain of gram-negative bacteria in the reduction of tellurite. Arch Microbiol. 173, 178–186 (2000). 10763749 10.1007/s002039900123

[b20] Zannoni D. Bacterial processing of metalloids as a tool in Biotechnology. J Biotechnol. 150, S52–S53 (2010).

[b21] Mohanty A., Liu Y., Yang L. & Cao B. Extracellular biogenic nanomaterials inhibit pyoverdine production in *Pseudomonas aeruginosa*: A novel insight into impacts of metal(loid)s on environmental bacteria. Applied Microbiology and Biotechnology. 99, 1957–1966 (2015). 25273177 10.1007/s00253-014-6097-5

[b22] Fleming A. & Young M. Y. The inhibitory action of potassium tellurite on coliform bacteria. J Pathol Bacteriol 51, 29–35 (1940).

[b23] Cummins J., Reen F. J., Baysse C., Mooij M. J. & O’Gara F. Subinhibitory concentrations of the cationic antimicrobial peptide colistin induce the pseudomonas quinolone signal in *Pseudomonas aeruginosa*. Microbiology 155, 2826–2837 (2009). 19477905 10.1099/mic.0.025643-0

[b24] Brazas M. D. & Hancock R. E. Ciprofloxacin induction of a susceptibility determinant in *Pseudomonas aeruginosa*. Antimicrob Agents Chemother 49, 3222–3227 (2005). 16048929 10.1128/AAC.49.8.3222-3227.2005 PMC1196232

[b25] Thompson D. K. *et al.* Proteomics reveals a core molecular response of *Pseudomonas putida* F1 to acute chromate challenge. BMC Genomics 11, 311 (2010). 20482812 10.1186/1471-2164-11-311 PMC2996968

[b26] Perez J. M. *et al.* Bacterial toxicity of potassium tellurite: unveiling an ancient enigma. PLoS One 2, e211 (2007). 17299591 10.1371/journal.pone.0000211 PMC1784070

[b27] Bebien M., Chauvin J. P., Adriano J. M., Grosse S. & Vermeglio A. Effect of selenite on growth and protein synthesis in the phototrophic bacterium *Rhodobacter sphaeroides*. Appl Environ Microbiol. 67, 4440–4447 (2001). 11571140 10.1128/AEM.67.10.4440-4447.2001 PMC93187

[b28] Alhede M. *et al.* Phenotypes of non-attached *Pseudomonas aeruginosa* aggregates resemble surface attached biofilm. PLoS One 6, e27943 (2011). 22132176 10.1371/journal.pone.0027943 PMC3221681

[b29] Borlee B. R. *et al.* *Pseudomonas aeruginosa* uses a cyclic-di-GMP-regulated adhesin to reinforce the biofilm extracellular matrix. Mol Microbiol. 75, 827–842 (2010). 20088866 10.1111/j.1365-2958.2009.06991.x PMC2847200

[b30] Rybtke M. T. *et al.* Fluorescence-Based Reporter for Gauging Cyclic Di-GMP Levels in *Pseudomonas aeruginosa*. Appl Environ Microbiol. 78, 5060–5069 (2012). 22582064 10.1128/AEM.00414-12 PMC3416407

[b31] Jacob-Dubuisson F., Locht C. & Antoine R. Two-partner secretion in Gram-negative bacteria: a thrifty, specific pathway for large virulence proteins. Mol Microbiol. 40, 306–313 (2001). 11309114 10.1046/j.1365-2958.2001.02278.x

[b32] Gottig N., Garavaglia B. S., Garofalo C. G., Orellano E. G. & Ottado J. A filamentous hemagglutinin-like protein of *Xanthomonas axonopodis* pv. citri, the phytopathogen responsible for citrus canker, is involved in bacterial virulence. PLoS One 4, e4358 (2009). 19194503 10.1371/journal.pone.0004358 PMC2632755

[b33] Serra D. O. *et al.* FHA-mediated cell-substrate and cell-cell adhesions are critical for *Bordetella pertussis* biofilm formation on abiotic surfaces and in the mouse nose and the trachea. PLoS One 6, e28811 (2011). 22216115 10.1371/journal.pone.0028811 PMC3245231

[b34] Irie Y. *et al.* Self-produced exopolysaccharide is a signal that stimulates biofilm formation in *Pseudomonas aeruginosa*. P Natl Acad Sci USA 109, 20632–20636 (2012). 10.1073/pnas.1217993109 PMC3528562 23175784

[b35] Klebensberger J., Birkenmaier A., Geffers R., Kjelleberg S. & Philipp B. SiaA and SiaD are essential for inducing autoaggregation as a specific response to detergent stress in *Pseudomonas aeruginosa*. Environ Microbiol. 11, 3073–3086 (2009). 19638175 10.1111/j.1462-2920.2009.02012.x

[b36] Sakuragi Y. & Kolter R. Quorum-sensing regulation of the biofilm matrix genes (pel) of *Pseudomonas aeruginosa*. Journal of bacteriology 189, 5383–5386 (2007). 17496081 10.1128/JB.00137-07 PMC1951888

[b37] Chew S. C. *et al.* Dynamic remodeling of microbial biofilms by functionally distinct exopolysaccharides. mBio. 5, e01536–01514 (2014). 25096883 10.1128/mBio.01536-14 PMC4128364

[b38] Yang L. *et al.* Distinct roles of extracellular polymeric substances in *Pseudomonas aeruginosa* biofilm development. Environ Microbiol 13, 1705–1717 (2011). 21605307 10.1111/j.1462-2920.2011.02503.x

[b39] Potrykus K. & Cashel M. (p)ppGpp: still magical? Annu Rev Microbiol. 62, 35–51 (2008). 18454629 10.1146/annurev.micro.62.081307.162903

[b40] West S. A., Diggle S. P., Buckling A., Gardner A. & Griffins A. S. The social lives of microbes. Annu Rev Ecol Evol S 38, 53–77 (2007).

[b41] Sun F. *et al.* Quorum-sensing agr mediates bacterial oxidation response via an intramolecular disulfide redox switch in the response regulator AgrA. Proc Natl Acad Sci U S A 109, 9095–9100 (2012). 22586129 10.1073/pnas.1200603109 PMC3384213

[b42] O’Toole G. A. & Stewart P. S. Biofilms strike back. Nature biotechnology 23, 1378–1379 (2005). 10.1038/nbt1105-1378 16273068

[b43] Hoiby N., Bjarnsholt T., Givskov M., Molin S. & Ciofu O. Antibiotic resistance of bacterial biofilms. International journal of antimicrobial agents 35, 322–332 (2010). 20149602 10.1016/j.ijantimicag.2009.12.011

[b44] Wang S. *et al.* The exopolysaccharide Psl-eDNA interaction enables the formation of a biofilm skeleton in *Pseudomonas aeruginosa*. Environ Microbiol Rep, 7, 330–340 (2015). 25472701 10.1111/1758-2229.12252 PMC4656019

[b45] Chen Y. *et al.* Multiple diguanylate cyclase-coordinated regulation of pyoverdine synthesis in *Pseudomonas aeruginosa*. Environ Microbiol Rep, In Press (2015). 10.1111/1758-2229.12278 25683454

[b46] Wu Y., Ding Y., Cohen Y. & Cao B. Elevated level of the second messenger c-di-GMP in *Comamonas testosteroni* enhances biofilm formation and biofilm-based biodegradation of 3-chloroaniline. Appl Microbiol Biotechnol. 99, 1967–1976 (2015) 25273178 10.1007/s00253-014-6107-7

[b47] Bertani G. Studies on lysogenesis. I. The mode of phage liberation by lysogenic Escherichia coli. J Bacteriol 62, 293–300 (1951). 14888646 10.1128/jb.62.3.293-300.1951 PMC386127

[b48] Starkey M. *et al.* *Pseudomonas aeruginosa* rugose small-colony variants have adaptations that likely promote persistence in the cystic fibrosis lung. J Bacteriol 191, 3492–3503 (2009). 19329647 10.1128/JB.00119-09 PMC2681918

[b49] O’Toole G. A. & Kolter R. Flagellar and twitching motility are necessary for *Pseudomonas aeruginosa* biofilm development. Mol Microbiol. 30, 295–304 (1998). 9791175 10.1046/j.1365-2958.1998.01062.x

[b50] Ng C. K. *et al.* Influence of outer membrane c-type cytochromes on particle size and activity of extracellular nanoparticles produced by *Shewanella oneidensis*. Biotechnol Bioeng. 110, 1831–1837 (2013). 23381725 10.1002/bit.24856

[b51] Wiegand I., Hilpert K. & Hancock R. E. Agar and broth dilution methods to determine the minimal inhibitory concentration (MIC) of antimicrobial substances. Nat Protoc. 3, 163–175 (2008). 18274517 10.1038/nprot.2007.521

[b52] Smale S. T. Beta-galactosidase assay. Cold Spring Harbor protocols 2010, pdb prot5423 (2010). 10.1101/pdb.prot5423 20439410

[b53] Flohr R. C., Blom C. J., Rainey P. B. & Beaumont H. J. Founder niche constrains evolutionary adaptive radiation. Proceedings of the National Academy of Sciences of the United States of America 110, 20663–20668 (2013). 24306929 10.1073/pnas.1310310110 PMC3870684

[b54] Liu Y., Yang L. & Molin S. Synergistic activities of an efflux pump inhibitor and iron chelators against *Pseudomonas aeruginosa* growth and biofilm formation. Antimicrob Agents Chemother 54, 3960–3963 (2010). 20566773 10.1128/AAC.00463-10 PMC2935009

[b55] Holloway B. W. & Morgan A. F. Genome organization in *Pseudomonas*. Annu Rev Microbiol. 40, 79–105 (1986). 3535656 10.1146/annurev.mi.40.100186.000455

[b56] West S. E., Schweizer H. P., Dall C., Sample A. K. & Runyen-Janecky L. J. Construction of improved Escherichia-Pseudomonas shuttle vectors derived from pUC18/19 and sequence of the region required for their replication in Pseudomonas aeruginosa. Gene 148, 81–86 (1994). 7926843 10.1016/0378-1119(94)90237-2

[b57] Starkey M. *et al.* Pseudomonas aeruginosa Rugose Small-Colony Variants Have Adaptations That Likely Promote Persistence in the Cystic Fibrosis Lung. J Bacteriol 191, 3492–3503 (2009). 19329647 10.1128/JB.00119-09 PMC2681918

[b58] Kirisits M. J., Prost L., Starkey M. & Parsek M. R. Characterization of colony morphology variants isolated from *Pseudomonas aeruginosa* biofilms. Appl Environ Microbiol. 71, 4809–4821 (2005). 16085879 10.1128/AEM.71.8.4809-4821.2005 PMC1183349

[b59] Hoang T. T., Karkhoff-Schweizer R. R., Kutchma A. J. & Schweizer H. P. A broad-host-range Flp-FRT recombination system for site-specific excision of chromosomally-located DNA sequences: application for isolation of unmarked *Pseudomonas aeruginosa* mutants. Gene 212, 77–86 (1998). 9661666 10.1016/s0378-1119(98)00130-9

[b60] Kessler B., de Lorenzo V. & Timmis K. N. A general system to integrate lacZ fusions into the chromosomes of gram-negative eubacteria: regulation of the Pm promoter of the TOL plasmid studied with all controlling elements in monocopy. Mol Gen Genet 233, 293–301 (1992). 1318499 10.1007/BF00587591

