# Supplementary material for: C-di-GMP regulates Pseudomonas aeruginosa stress response to tellurite during both planktonic and biofilm modes of growth
Source: Sci Rep. 2015 May 20;5:10052. doi: 10.1038/srep10052 (PMC4438720; doi:10.1038/srep10052)
Supplement: Supplementary Information [file srep10052-s1.pdf]

C-di-GMP regulates *Pseudomonas aeruginosa* stress response to tellurite during both planktonic and biofilm modes of growth

Song Lin Chua [1,2], Krishnakumar Sivakumar [1,3], Morten Rybtke [4], Mingjun Yuan[1], Jens Bo Andersen [4], Thomas E. Nielsen [1,8], Michael Givskov [1,4], Tim Tolker-Nielsen [4], Bin Cao [1,5*], Staffan Kjelleberg [1,6], Liang Yang [1,7*]

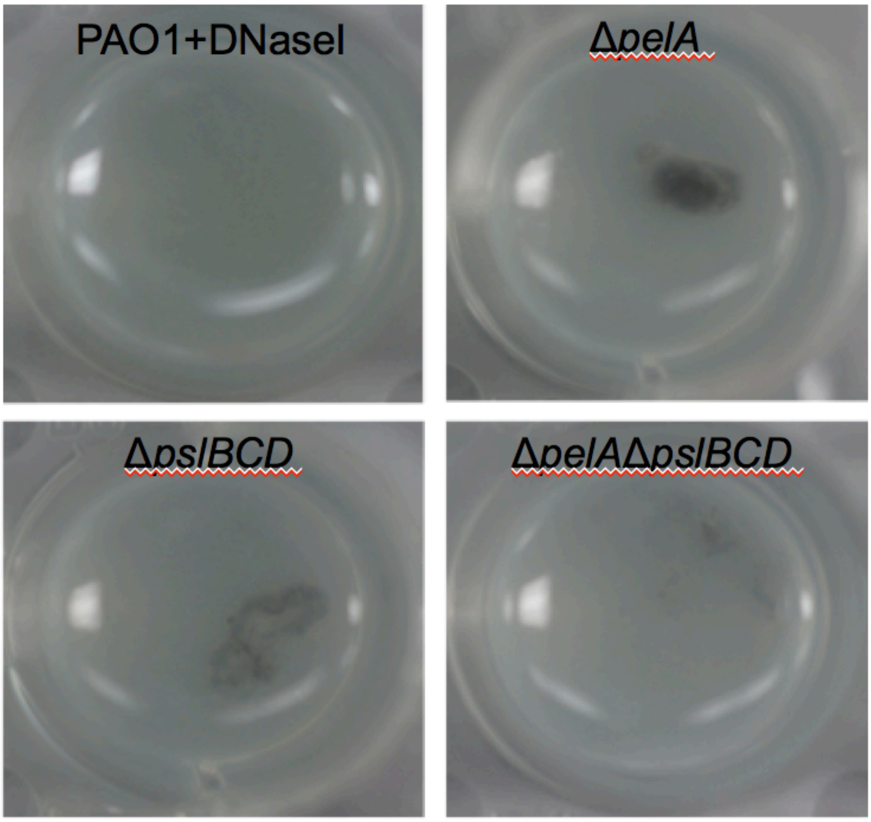

Figure S1. Aggregates formed by *P. aeruginosa* wild-type PAO1strain and the polysaccharide mutants in ABTGC medium with 10 µg/ml TeO_3^{2-} after 1 day under shaking condition. 10 µg/ml DNaseI was added to the PAO1 cultures. Representative image from triplicate experiments was shown for each condition.

Table S1 Up regulated proteins by tellurite

Gene number and protein function	Peptides(95%)	%Cov(95)	+Tel/-Tel	PVal
PA0572|hypothetical protein	250	82.88	99.08319	5.04E-05
PA2402|probable non ribosomal peptide synthetase	16	4.914	35.31832	0.008042
PA2424|PvdL	42	12.14	30.76097	6.62E-08
PA2760|OprQ	24	39.53	28.84031	0.012788
PA0563|hypothetical protein	6	12.98	28.84	0.0127
PA4225|pyochelin synthetase	2	24.43	28.31392	0.007283
PA4624|cyclic diguanylate regulated TPS partner B, CdrB	27	20.07	25.8226	4.17E-06
PA4226|dihydroaeruginoic acid synthetase	7	36.93	25.8226	9.06E-05
PA0999|3-oxoacyl-[acyl-carrier-protein]synthase III	56	11.57	25.11886	6.70E-06
PA3327|probable non ribosomal peptide synthetase	2	17.3	23.7684	0.039298
PA2386|L-ornithineN5-oxygenase	42	38.15	23.12065	8.48E-07
PA2399|pyoverdine synthetase D	21	25.2	22.69865	0.002347
PA4224|pyochelin biosynthetic protein PchG	29	20.92	22.49055	0.001658
PA0090|ClpV1	6	15.63	22.49055	0.000166
PA2400|PvdJ	11	7	20.89296	0.00982
PA4661|LipidA 3-O-deacylase	13	48.55	19.23092	0.023034
PA3038|probable porin	15	13.78	18.03018	8.10E-07
PA2398|ferripyoverdine receptor	14	50.18	15.99558	0.002339
PA2853|Outermembrane lipoprotein OprI precursor	5	45.78	14.99685	0.008247
PA3483|hypothetical protein	97	18.42	14.58814	0.002669
PA1742|Glutamineamido transferase class I	4	15	13.80384	0.018477
PA3335|hypothetical protein	58	16.8	13.67729	2.24E-13
PA2815|probable acyl-CoA dehydrogenase	26	36.56	13.67729	0.047206
PA3329|hypothetical protein	33	26.24	12.13389	7.29E-12
PA1271|probable tonB-dependent receptor	4	6.331	12.02264	0.005989
PA0079|TssK1	4	9.459	11.80321	0.009242
PA4221|Fe(III)-pyochelin outer membrane receptor precu	2	54.86	11.69499	0.002462
PA0595|organic solvent tolerance protein OstA precursor	3	24.78	11.37627	0.0013
PA2391|probable outer membrane protein precursor	53	45.36	10.86426	1.01E-08
PA3724|elastase LasB	16	35.54	10.86426	0.010083
PA0427|Major intrinsic multiple antibiotic resistance efflu	3	37.53	10.47129	0.006546
PA2366|uricase PuuD	14	6.478	10.18591	2.91E-07
PA3344|ATP-dependentDNA helicase RecQ	3	3.371	9.908319	0.034625
PA0973|Peptidoglycan associated lipoprotein OprL precu	92	46.43	9.817479	4.44E-16
PA0958|Basicamino acid, basic peptide and imipenem ou	84	46.73	9.817479	0.003556
PA3186|Glucose/carbohydrate outer membrane porin Op	6	38.11	9.727472	0.007463
PA2302|AmbE	22	3.861	9.120109	0.003599
PA2330|hypothetical protein	3	15.77	9.120109	0.015323
PA3408|Heme uptake outer membrane receptor HasR pr	5	14.48	8.709636	0.003476
PA5016|dihydrolipoamide acetyltransferase	18	48.26	8.472274	3.57E-08
PA1061|conserved hypothetical protein	63	67.4	8.317637	0.005835
PA3790|Putative copper transport outer membrane porin	16	54.08	8.090959	0.013769
PA3836|hypothetical protein	4	48.31	8.090959	0.002906
PA3728|hypothetical protein	4	1.833	8.016781	0.047031
PA1377|conserved hypothetical protein	2	68.36	8.016781	0.003154
PA3729|conserved hypothetical protein	376	6.686	7.870458	7.12E-09
PA0807|AmpDh3	2	51.76	7.798301	0.009569
PA3194|phosphogluconate dehydratase	36	49.84	7.244359	0.000351

PA3332|conserved hypothetical protein	27	37.59	7.112135	7.07E-06
PA0622|probable bacteriophage protein	26	48.96	6.668067	6.88E-07
PA4792|conserved hypothetical protein	12	50.8	6.426877	0.007643
PA0041|probable hemagglutinin	7	19.77	6.309574	0.008558
PA4676|probable carbonic anhydrase	4	68.37	6.309574	0.023276
PA4015|conserved hypothetical protein	36	51.66	6.251727	0.01664
PA0084|TssC1	9	17.47	6.251727	0.000131
PA1338|gamma-glutamyltranspeptidaseprecursor	31	19.03	6.025596	1.60E-08
PA4694|ketol-acidreductoisomerase	45	65.98	6.025596	0.022332
PA3238|hypothetical protein	25	15.67	5.754399	4.86E-06
PA1579|hypothetical protein	19	56.44	5.597576	0.008136
PA0662|N-acetyl-gamma-glutamyl-phosphatereductase	57	76.74	5.445026	5.26E-06
PA5076|probable binding protein component of ABC tran	14	58.27	5.345644	0.00502
PA4475|conserved hypothetical protein	3	36.88	5.296635	0.024291
PA4732|glucose-6-phosphateisomerase	9	32.31	5.248075	0.004052
PA4483|Glu-tRNA(Gln) amido transferase subunit A	4	60.74	5.152287	0.002782

Table S2 Down regulated proteins by tellurite

Gene number and protein function	Peptides(95%)	%Cov(95)	+Tel/-Tel	PVal
PA0141|conserved hypothetical protein	11	34.23	0.025823	0.000204
PA4687|ferric iron-binding periplasmic protein HitA	11	30.45	0.025823	0.000294
PA4352|conserved hypothetical protein	36	64.34	0.031333	8.93E-05
PA2951|electrontransfer flavoprotein alpha-subunit	52	73.79	0.039446	0.004405
PA4064|probable ATP-binding component of ABC transpo	7	55.56	0.048753	0.000648
PA4263|50S ribosomal protein L3	7	37.44	0.060256	0.006212
PA0430|5,10-methylenetetrahydrofolate reductase	8	42.76	0.060814	0.025942
PA1586|dihydrolipoamide succinyltransferase (E2 subunit	35	62.35	0.061376	0.002073
PA5015|pyruvate dehydrogenase	46	36.96	0.066069	8.21E-12
PA4671|probable ribosomal protein L25	24	79.9	0.06792	1.38E-05
PA1245|AprX	75	60.14	0.068549	0.0008
PA4244|50S ribosomal protein L15	7	40.97	0.069183	0.013156
PA1171|probable transglycolase	5	20.6	0.071779	0.023444
PA5554|ATP synthase beta chain	95	78.17	0.074473	5.58E-06
PA5173|carbamate kinase	6	23.23	0.07656	0.014051
PA3005|beta-N-acetyl-D-glucosaminidase	3	8.133	0.07656	0.018277
PA5556|ATP synthase alpha chain	72	53.5	0.080168	0.00031
PA0395|twitching motility protein PilT	10	38.37	0.087096	0.001232
PA2967|3-oxoacyl-[acyl-carrier-protein]reductase	9	39.27	0.087096	0.023841
PA4277|elongation factor Tu	211	70.53	0.087902	0.000138
PA5171|arginine deiminase	80	69.38	0.090365	6.57E-10
PA4587|cytochrome c551 peroxidase precursor	13	47.11	0.093756	0.013287
PA0372|probable zinc protease	4	17.2	0.097275	0.012433
PA1800|trigger factor	13	38.76	0.099083	0.00019
PA1789|hypothetical protein	13	39.02	0.099083	0.014599
PA3637|CTP synthase	16	35.42	0.100925	2.12E-05
PA1923|hypothetical protein	26	24.04	0.105682	0.004861
PA4639|hypothetical protein	8	45.13	0.105682	0.001514
PA3263|conserved hypothetical protein	6	26.14	0.105682	0.009988
PA4756|carbamoylphosphate synthetase large subunit	18	18.73	0.108643	0.00092
PA4260|50S ribosomal protein L2	12	30.4	0.109648	0.002423

PA4170|hypothetical protein	8	34.19	0.113763	0.047994
PA4771|L-lactate dehydrogenase	8	32.02	0.115878	0.001434
PA3187|probable ATP-binding component of ABC transpc	11	23.06	0.120226	0.023461
PA5427|alcohol dehydrogenase	17	52.92	0.121339	0.000618
PA2744|threonyl-tRNAsynthetase	7	17.5	0.125893	0.037254
PA5215|glycine-cleavagesystem protein T1	3	10	0.12942	0.025444
PA4063|hypothetical protein	6	25	0.130617	0.019117
PA3653|ribosome recycling factor	8	42.7	0.131826	0.003174
PA0956|prolyl-tRNAsynthetase	19	37.65	0.139316	4.70E-06
PA2393|probable dipeptidase precursor	13	33.93	0.140605	0.001438
PA0764|negative regulator for alginate biosynthesis MucI	7	30.06	0.140605	0.021213
PA1546|oxygen-independentcoproporphyrinogen III oxida	4	11.96	0.140605	0.012304
PA0887|acetyl-coenzymeA synthetase	21	33.95	0.144544	0.000498
PA0337|phosphoenolpyruvate-protein phosphotransfera:	9	15.55	0.145881	0.000231
PA2642|NADH dehydrogenase I chain G	32	33.48	0.149969	2.91E-05
PA1459|probable methyltransferase	5	18.75	0.155597	0.023353
PA2290|glucose dehydrogenase	39	43.71	0.157036	0.001152
PA0459|probable ClpA/B protease ATP binding subunit	21	28.35	0.158489	5.60E-06
PA5054|heat shock protein HslU	6	16.55	0.159956	0.001317
PA5172|ornithinecarbamoyl transferase, catabolic	83	65.48	0.167494	1.11E-08
PA0296|Glutamylpolyamine synthetase	4	13.1	0.169044	0.01579
PA2952|electrontransfer flavoprotein beta-subunit	26	74.3	0.172187	0.000366
PA0668|tyrosyl-tRNAsynthetase 2	7	19.05	0.175388	0.029985
PA4310|chemotactic transducer PctB	14	23.21	0.177011	0.023584
PA4542|ClpB protein	15	25.29	0.187068	0.000668
PA4932|50S ribosomal protein L9	7	56.76	0.187068	0.007105
PA4386|GroESprotein	4	52.58	0.187068	0.031502
PA0265|succinate-semialdehydedehydrogenase	34	60.46	0.190546	3.84E-06
PA3183|glucose-6-phosphate1-dehydrogenase	20	56.24	0.192309	0.000856
PA0295|probable periplasmic polyamine binding protein	10	35.41	0.192309	0.016069
PA5493|DNA polymerase I	13	18.4	0.195884	0.001282
PA1787|aconitate hydratase 2	46	36.13	0.199526	0.001423
PA4423|conserved hypothetical protein	14	36.59	0.199526	3.90E-05
PA2968|malonyl-CoA-[acyl-carrier-protein]transacylase	13	44.23	0.199526	0.020452
